# 肿瘤自身抗体在肺癌早期诊断中的作用

**DOI:** 10.3779/j.issn.1009-3419.2010.09.12

**Published:** 2010-09-20

**Authors:** 志浩 吴

**Affiliations:** 300052 天津，天津医科大学总医院，天津市肺癌研究所，天津市肺癌转移与微环境重点实验室 Tianjin Key Laboratory of Lung Cancer Metastasis and Tumor Microenviroment, Tianjin Lung Cancer Institute, Tianjin Medical University General Hospital, Tianjin 300052, China

肺癌是当今世界上对人类健康与生命危害最大的恶性肿瘤之一，其死亡率居各类恶性肿瘤所致死亡的首位。世界卫生组织(World Health Organization, WHO)预测，到2025年中国每年将有超过一百万新发肺癌患者^[1]^。最新统计数据^[2, 3]^显示，肺癌的总5年生存率仅10%-16%，而Ⅰ期患者的5年生存率可达65%-75%。虽然目前临床上已经联合影像学技术、痰细胞学检测、纤维支气管镜等多种检测手段，但对无症状早期肺癌的诊断效果甚微，多数患者在就诊时已发展至晚期。因此，寻找有效的早期诊断技术和方法对于延长患者生命至关重要。

近年来，许多研究^[4-9]^表明肿瘤在发生早期即可激发宿主体内产生体液免疫，在多种肿瘤包括肺癌患者的血清中，均可检测到针对肿瘤相关抗原(tumour-associated antigens, TAAs)的自身抗体的存在。因此，对肿瘤早期抗原和抗体的免疫检测逐渐成为肿瘤早诊研究领域的一个热点问题。本文综合了肿瘤体液免疫反应的发现和作用机理，并对肿瘤自身抗体作为标志物在肺癌早诊中的作用以及其鉴定方法进行了阐述。

## 1 肿瘤抗原与肺癌体液免疫的关系

在肿瘤免疫学的研究历史中，有两个主要问题曾一度是人们争论的焦点：是否存在肿瘤抗原？如果存在，这些抗原是否能并如何被机体免疫系统识别？20世纪初，Paul Ehrlich根据在动物个体间进行肿瘤移植时，移植瘤能自行萎缩消退的现象，第一次提出了肿瘤抗原及肿瘤免疫的概念。1966年，Baldwin^[10]^首次证实了机体免疫系统对自发肿瘤的鉴别和排斥反应，从而奠定了肿瘤抗原研究的理论基础。20世纪70年代初免疫监视概念正式被提出，认为在肿瘤发生早期，机体的免疫系统可以及时识别癌细胞，并进行清除或加以抑制，该理论为癌症的早期检测提供了新的思路。免疫监视理论成立的前提是有肿瘤抗原的存在，而机体正是依靠肿瘤抗原来识别肿瘤细胞^[11]^。

肿瘤抗原是指细胞癌变过程中出现的新抗原及过度表达的抗原物质的总称。这些由于基因过表达，突变的蛋白产物或变体、异常降解蛋白可以作为肿瘤抗原诱导机体产生免疫应答。p53是目前研究最广泛的肿瘤抗原之一。在所有恶性肿瘤中，50%以上会出现*p53*基因的突变^[12]^，研究^[13, 14]^显示肺癌患者可发生多种*p53*基因的突变，包括错义突变、终止密码子突变和移码突变，但只有错义突变产生过度表达蛋白，p53突变蛋白水平的增加不但具有免疫原性、引发免疫反应，而且这些过度表达蛋白发生了功能改变，增强了与抗体产物结合的稳定性。而经过转录后修饰的肿瘤抗原也可以诱导免疫反应，如糖基化、磷酸化、氧化和蛋白水解酶切等转录后修饰过程是通过产生新表位和增强自身表位呈递和对主要组织相容性复合物或T细胞受体的亲和性来激发免疫应答的，进而产生针对肿瘤抗原免疫原性表位的自身抗体^[15]^。Hoagland等^[16]^通过比较非小细胞肺癌患者和正常对照血清中结合珠蛋白的不同形式的糖基化水平，发现二者具有明显统计学差异，而且糖基化蛋白的浓度与肿瘤分期还具有一定相关性。目前，修饰抗原引发体液免疫的机制还不完全清楚，特别是发现许多TAAs属于细胞内蛋白^[17]^。其中一种假说认为肿瘤细胞的异常死亡导致修饰的胞内蛋白从肿瘤细胞中释放出来，暴露于机体的免疫系统中，这种反复的异常肿瘤细胞死亡可导致修饰的胞内蛋白持续暴露，同时肿瘤细胞死亡过程中所释放的蛋白酶可使蛋白隐藏的自身表位暴露进一步引发免疫反应^[18, 19]^。

此外，一些常用的肿瘤抗原如癌胚抗原(carcinoembryonic antigen, CEA)、细胞角蛋白19片段抗原(cytokeratin fragment antigen 21-1, CYFRA21-1)、粘蛋白1抗原(mucin 1 antigens, M1A)、组织多肽特异性抗原(tissue polypeptide specific antigen, TPS)等已被广泛应用于肺癌临床检测中，但诊断的敏感度均不够理想。

总的来说，机体产生免疫原性肿瘤抗原的机制可归结为以下6种：基因突变；细胞癌变过程中原本不表达的基因被激活；蛋白合成过程的某些环节发生异常(如糖基化异常、磷酸化异常、蛋白水解酶切等导致蛋白质特殊产物的产生)；胚胎时期抗原或分化抗原的异常、异位表达；某些基因产物尤其是信号转导分子的过度表达；外源性基因(如病毒基因)的表达。这些肿瘤抗原可作为评价早期诊断和患者预后的分子标记，鉴定这些免疫标记物，并探讨其功能和对肿瘤形成机制的影响可能有助于揭示肿瘤形成过程中的早期分子事件^[20]^。

## 2 肿瘤自身抗体在肺癌早期检测中的作用

大量研究^[8, 21, 22]^表明，血清中的肿瘤抗原等标志物能诱导机体产生自身抗体，在肿瘤发生还未能被临床检查手段检测到的早期，机体免疫系统就可监测到低水平表达的肿瘤抗原的存在，并引发免疫反应，产生大量的抗体，起到有效的生物信号放大作用，虽然这些天然抗体的确切来源至今还未有定论，但有一种解释是其来源于自体反应或非成熟B淋巴细胞，例如在乳腺髓样癌中，肌动蛋白暴露于肿瘤细胞表面引发B淋巴细胞浸润，导致大范围的抗体反应而使肿瘤细胞坏死增加^[23]^。所以理论上利用肿瘤自身抗体检测肿瘤的特异性和敏感性均比利用肿瘤抗原检测肿瘤要高得多^[24]^，是一种非常有前景的肿瘤检测标志物。

血清抗体比其它血清蛋白在肺癌的诊断中占优势还体现在以下几个方面。第一，可以对肿瘤进行早期诊断，便于早期治疗，提高治愈率。多项研究^[25, 26]^表明在影像学检查确诊实体癌数月至数年前即可检测到自身抗体的存在，甚至有报道^[27]^显示在肺癌确诊前5年就可检测到血清中有针对肿瘤抗原的抗体。Qiu等^[28]^用肺癌细胞系A549的分离蛋白制成芯片，对肺癌确诊前1年内的血清样本和正常对照血清样本进行检测，发现annexin I、14-3-3 theta和LAMR1的自身抗体在无症状期就已经存在，提示自身抗体检测可作为肺癌高危人群筛查早诊的一种方法。第二，自身抗体比相应肿瘤抗原滴度高，针对单一抗原的自身抗体通过免疫反应大量扩增，在血清中大量白蛋白存在干扰的情况下，较其它标志物更易检测到^[29]^。第三，标本易获得，检测结果相对稳定。肿瘤抗原一旦被分泌入血，可能很快地被降解或清除，而自身抗体不像其它多肽一样易受蛋白酶水解作用，可在相当长一段时间内在血清中稳定、持续存在，并且自身抗体的半衰期很长，大约是7天，每小时的波动很小，理化性质稳定，在-80 ℃长期保存对其活性几乎无影响^[30]^。第四，其操作所用的试剂和技术简便易行，重复性好，通过常规的酶联免疫吸附试验(ELISA)或酶联免疫分析(EIA)就可以检测。

## 3 肿瘤自身抗体联合检测在肺癌早期诊断中的应用

肿瘤发生是一个多基因、多步骤的癌变过程，仅靠一个指标进行诊断，常常会导致假阳性和假阴性，所以，单个肿瘤自身抗体检测必然存在一些局限性，影响肺癌的临床诊断。例如，许多在肿瘤产生和发展过程中起重要作用的热休克蛋白(HSPs)经常在肿瘤中呈过表达状态，HSP70等的抗体在多种肿瘤中均可被检测到^[31]^。一般情况下，同种肿瘤可异常地产生一种或多种肿瘤自身抗体，而不同肿瘤或同种肿瘤的不同组织类型既可检出共同的肿瘤抗体，也可检出不同的肿瘤抗体。糖类抗原12-5(CA12-5)虽然是公认的卵巢癌相关抗原，但由于肺组织具有与CA-125相同的抗原特性，所以其抗体在肺癌血清中的阳性检出率也相当高^[32]^。这是由肿瘤细胞生物学特性的复杂性及多态性、不同肿瘤病理类型的差异、同种病理类型肿瘤细胞的异质性和肿瘤细胞基因型及细胞表型的差异等多种因素所决定的。

近年来的研究倾向于针对联合抗体谱进行癌症检测。Chapman等^[33]^运用ELISA对肺癌(包括非小细胞肺癌和小细胞肺癌)患者与正常对照人群血清中针对7种肿瘤抗原(p53、c-myc、HER2、NY-ESO-1、CAGE、MUC1和GBU4-5)的血清抗体进行检测，结果显示单一抗体检出阳性率在5%-36%之间，诊断的特异性达到96%-100%，而7种抗体联合检测的敏感性可达76%，特异性为92%。Zhong等^[34]^将肺癌噬菌体文库中所筛选出的免疫原性噬菌体表达蛋白构建成一个蛋白表达芯片，在大量患者和正常对照组血清中进行鉴定、分析后，选出5个表达蛋白标志物组成联合诊断模型，其对非小细胞肺癌联合检测的敏感性达到90%，特异性为95%。这些研究充分说明在进行肿瘤标志物单项检测时，特异性较高，但敏感性和准确性较低；联合检测后，虽然特异性有所降低，但敏感性和准确性均有很大的提高，如果在实际应用中进行优化组合，可显著提高肺癌早期诊断率，具有很好的临床应用价值。

## 4 肿瘤自身抗体的鉴定方法

成功的肿瘤血清抗体标志物鉴定方法依赖于高通量的筛选策略，随着蛋白质组学的发展，相比于最初的单相的十二烷基磺酸钠一聚丙烯酰胺凝胶电泳(SDS/PAGE)蛋白分离技术或ELISA而言，目前改进的蛋白组学方法(如蛋白质芯片等)所筛选的肿瘤血清抗体标志物具有更高的诊断和临床应用价值，操作也更加简便高效^[30, 35]^。下面介绍几种基于抗原抗体免疫基础上的高通量蛋白组学鉴定方法。

### 4.1 重组cDNA表达文库血清学分析

1995年，Sahin小组^[36]^首次建立重组cDNA表达文库血清学分析(serological analysis of recombinant cDNA expression library, SEREX)技术。它将分子克隆技术和利用患者血清对肿瘤细胞的自体分型技术结合在一起，不仅可检测抗体反应，还可通过患者自体血清中抗原抗体反应，直接从分子水平确定抗原特性。

SEREX的基本工作流程是：提取肿瘤细胞或组织的mRNA，构建cDNA表达文库，用含高滴度抗体的患者血清筛选cDNA表达文库，经过2轮-3轮复筛后阳性单克隆得以富集纯化。对所获得的阳性克隆进行序列测定，然后对其进行生物信息学分析，再进一步从分子和蛋白水平这些基因的结构和功能进行研究，同时用已知抗原检测体内相应抗体的浓度，定量分析肿瘤特异性抗体及其临床意义。

随着研究的不断深入，迄今为止，已有超过2 300种肿瘤抗原基因通过SEREX技术被识别鉴定，并进入肿瘤免疫组数据库(http://ludwig-sun5.unil.ch/CancerImmunomeDB/)，其中70%为已知基因，这些能够被机体免疫系统识别的基因产物主要包括转录和翻译调控因子、代谢酶和细胞表面受体等，为肿瘤早期检测和免疫治疗提供了广泛的分子靶点。Yue等^[37]^使用非小细胞肺癌血清对噬菌体展示文库进行筛选，发现15个肺癌相关抗原和3个肺癌相关抗原的候选基因。这种方法较之于其它筛选方法，具有分析全面(全长cDNA，涵盖了由肿瘤基因编码的大多数蛋白、还可以鉴定出T细胞依赖性抗原^[38]^)、高效(一次筛选过程中鉴定出多个抗原)、方便(不需要特异CTL和瘤细胞的体外建株)等优点。但同时也存在一些局限性，比如不适用于鉴定具有翻译后修饰功能的肿瘤相关抗原^[39]^；过高表达基因编码抗原(如癌-睾丸抗原)导致此方法筛选出现固有偏倚，而使低表达的抗原无法筛选出来^[40]^；庞大的工作量不适用于对多例患者血清进行分析。

### 4.2 噬菌体展示技术

噬菌体展示技术是表达蛋白的表现型和编码基因的基因型之间的完美结合。其原理是以噬菌体为载体，将编码外源蛋白或多肽的基因片段定向插入到噬菌体的外壳蛋白基因区，使外源蛋白或多肽通过与噬菌体外壳蛋白融合而表达并展示于噬菌体表面，被展示的蛋白或多肽可保持相对独立的空间结构和生物活性，进而通过亲和富集等方法筛选表达特异蛋白或多肽的噬菌体^[41]^。

目前常用的噬菌体展示系统主要有丝状噬菌体展示系统、λ噬菌体展示系统、T4噬菌体展示系统和T7噬菌体展示系统。由于噬菌体的这种特性使此技术生物淘洗过程更加省时、省力，目前多采用G蛋白珠(protein-G beads)来进行生物淘洗，通过亲和-离心-洗脱-扩增的循环步骤，筛选富集到与患者血清抗体特异结合的噬菌体克隆，这些阳性克隆可以点在玻璃或硝酸纤维素膜上组成微阵列，供大量样本高通量检测^[42]^。但这种方法虽然比SEREX更加高通量，但两者在筛选蛋白性质方面有相同的局限性。

### 4.3 血清蛋白质分析技术

血清蛋白质分析技术(serological proteome analysis, SERPA)技术是双向凝胶电泳(2-DE)、蛋白印迹(Western blot)和质谱技术相结合产生的一种高通量的新技术^[43]^，此技术不必构建表达文库，可分析大量患者的血清样品，同时可统计肿瘤抗体的发生频率，更重要的是可以发现经过各种翻译后修饰的蛋白抗原。故此技术一经发明，立即被广泛应用于肺癌、乳腺癌等多种肿瘤抗原的筛选及鉴定^[44, 45]^。

SERPA的基本原理是利用双向电泳分离肿瘤组织或细胞的总蛋白后将其转膜，再与肿瘤患者的血清免疫杂交而显色，通过质谱鉴定双向凝胶上对应的反应点而确定肿瘤抗原。另一种与此技术相似的方法是多重亲和蛋白谱分析(multiple affinity protein profiling, MAPP)，但其自动化程度更高。这两种方法的缺陷主要与2-DE的技术局限性有关，很难检测到低丰度蛋白、低分子量、疏水性及不可溶性跨膜蛋白等。

### 4.4 蛋白质微阵列

蛋白质微阵列(protein microarray)又称为蛋白质芯片(protein chip)，是通过微加工技术和微电子技术在固体表面构建的微型生物化学分析系统，可作为一种高通量肿瘤自身抗体分析的有力工具。

生物蛋白芯片技术是将位置及序列为已知的大量蛋白(纯化或重组的蛋白、肿瘤组织和细胞裂解蛋白等)、多肽分子、酶、抗原、抗体以预先设计的方式固定在载体上组成密集的分子排列，这个载体可以是二维的(如玻片、硝酸纤维素膜和微孔板等)或三维的(如微珠和纳米颗粒)，当标记的靶分子与芯片上的探针分子结合后，通过激光共聚焦扫描或光耦合元件(charge coupled device, CCD)对标记信号的强度进行检测^[46, 47]^。

蛋白质芯片技术与传统检测方法相比，芯片技术可以达到一次试验同时检测多种疾病或分析多种生物样品的目的；使用样品用量小；灵敏度和可靠性极高；自动化程度高等优点^[48]^。Madoz-Gúrpide等^[49]^采用肿瘤细胞系的天然蛋白微阵列分析肺癌细胞系A549分离蛋白与肺癌患者及正常对照人群血清的免疫反应，鉴定出8种蛋白可与肺癌患者血清发生反应，而与正常人血清不发生反应(*P*＜0.01)。此外，还发现肺癌患者血清中抗PGP9.5自身抗体明显升高，提示其有可能用于肺癌的诊断。现在已有商业化蛋白质芯片问世并应用于肿瘤诊断研究，由美国Invitrogen公司生产的蛋白质芯片可一次性检测8 000种重组蛋白^[50]^。目前，蛋白质芯片技术在早期诊断肿瘤标志物的发现和筛查、监测肿瘤进程和个体化治疗反应等方面已取得长足进展。但在成本、数据分析等方面的不足还在改进中，有待真正实现低成本、标准化、规模化。

## 5 结论与展望

肿瘤的早期诊断是提高治愈率的关键，而取材简单、无创伤、价格低的血清学检测方法逐渐受到临床应用研究学者的广泛重视。体液免疫为肺癌的早发现、早诊断提供了理论依据，由于机体免疫系统对低表达的肿瘤抗原信号可进行放大作用，使自身抗体的检测比抗原检测更加有效，而多抗体联合检测更能提高检测的敏感性，结合高通量蛋白组学研究方法，为肿瘤标志物的发现和鉴定提供了有利手段。但迄今为止所发现的标志物与各项临床指标(如组织类型、分期等)的关系并不密切，其临床应用前景不容乐观^[51]^。因此，未来的研究方向也许需要广泛的多中心合作、多种检测手段来评估候选标志物在敏感性、特异性和诊断准确性方面价值，以建立高灵敏度和准确率的肿瘤早期诊断预警的多分子判别体系。
